# Gun-Shot Wound of the Brain

**Published:** 1873-12

**Authors:** P. W. Van Peyma

**Affiliations:** Resident Physician Buffalo General Hospital


ART. II.—*Gun Shot Wound of Brain—Post-mortem.* By P. W. VAN PEYMA, M. D., Resident Physician Buffalo General Hospital.

That the following case of gun-shot injury of the brain is one of unusual interest, and that it is worthy of record, will be admitted by all:

On the 13th of November a man was brought to the Buffalo General Hospital in a comatose state. The antecedent history of his case was very incomplete. He had been found on the previous evening in an insensible condition, and had subsequently roused sufficiently to give his name, which he said was Matthias Miller. This was all that was known when admitted. He presented the symptoms of cerebral disturbance. An extended record of his symptoms is preserved; but as the peculiar interest of this case does not depend on these, I shall omit them, merely saying that they were those usually present in the class of cases to which this one belonged. It will, however, be proper to say that subsequent to his admission the patient was at one time sufficiently roused to give his name and age, which latter was fifty. He had two slight bruises of the scalp, which did not appear to me to be recent in character. One of these was situated over the superior angle of the occipital, the other over the right temporal region. With these exceptions nothing was noticed to show external violence. He died on the sixth day after admission.

In view of the general obscurity of the case an autopsy was determined upon. The meninges were carefully examined, and on the right side they were found considerably congested. On removing the brain a collection of pus was found at its base, extending from the medulla oblongata forward. The lateral ventricles were also found filled with a purulent collection. At this moment, as the incisions were being extended, something was heard to fall on the tray on which the brain was lying. To our utter amazement this was found to be a bullet. The ball, which was of small size and considerably flattened, had been liberated by the knife. The conviction was forced upon us that the external opening through which the ball had passed had been overlooked during the life of the patient, and that this was the real cause of death. But our astonishment was increased when, after a careful examinatson of the surface, no opening could be found. As a last resort, the cranium was examined from the interior, and on the anterior surface, above and a little to the right of the left orbit, was found a fracture of the frontal bone, the internal table of which was extensively fissured. With this as a guide, we again made search for the external aperature, and again failed in finding an opening, but did find a discoloration of the skin over the seat of the fracture of a lead color, circular in shape and the size of the ball. There was not the least sign of a wound or the slightest scar. The wound, which must have existed, had healed perfectly, and left nothing but this leaden discoloration to show its former presence. This discoloration, which even after death was but slightly noticeable, must have been less so during life. The course of the ball through the brain could still be traced by a probe to the place where it had lodged near the anterior surface of the medulla. The opening in the bone was filled in with a gelatinous material through which a tenaculum passed readily.

The entire want of the previous history of the case is much to be regretted. How long the ball had been there, or what the patient's health was during this time, are questions which must as yet remain unanswered. Sufficient time had certainly elapsed to allow the external wound to heal.

It is to be hoped that the investigations now in progress may determine these points. The number of cases now on record of temporary recovery from these injuries, where the ball has been allowed to remain, is exceedingly limited.

